# Navigating therapeutic challenges in VEXAS syndrome: exploring IL-6 and JAK inhibitors at the forefront

**DOI:** 10.1186/s10020-024-00922-8

**Published:** 2024-09-17

**Authors:** Xiao Xiao Li, Wen Hui Huang, Xiao Bin Yang, Qi Lin Yang, Yu Zheng, Yong Bao Huo, Ting Ting Xie, Cheng Hui Huang, Shui Lian Yu

**Affiliations:** 1 Department of Rheumatology, The Second Affiliated Hospital of Guangzhou Medical University, Guangzhou Medical University, Guangzhou, Guangdong People’s Republic of China; 2 Department of Otolaryngology, The Second Affiliated Hospital of Guangzhou Medical University, Guangzhou Medical University, Guangzhou, Guangdong China; 3 Department of Critical Care Medicine, The Second Affiliated Hospital of Guangzhou Medical University, Guangzhou Medical University, Guangzhou, Guangdong China; 4 Department of Urology, The Second Affiliated Hospital of Guangzhou Medical University, Guangzhou Medical University, Guangzhou, Guangdong China; 5 Department of Gastroenterology, The Second Affiliated Hospital of Guangzhou Medical University, Guangzhou Medical University, Guangzhou, Guangdong China

**Keywords:** VEXAS syndrome, IL-6 Inhibitors, JAK–STAT, Treatment, Tocilizumab

## Abstract

VEXAS syndrome, an uncommon yet severe autoimmune disorder stemming from a mutation in the UBA1 gene, is the focus of this paper. The overview encompasses its discovery, epidemiological traits, genetic underpinnings, and clinical presentations. Delving into whether distinct genotypes yield varied clinical phenotypes in VEXAS patients, and the consequent adjustment of treatment strategies based on genotypic and clinical profiles necessitates thorough exploration within the clinical realm. Additionally, the current therapeutic landscape and future outlook are examined, with particular attention to the potential therapeutic roles of IL-6 inhibitors and JAK inhibitors, alongside an elucidation of prevailing limitations and avenues for further research. This study contributes essential theoretical groundwork and clinical insights for both diagnosing and managing VEXAS syndrome.

## Introduction

VEXAS syndrome, an infrequent and grave autoimmune malady arising from a *UBA1* gene mutation. Initially proposed in 2020 (Beck et al. 2020), the syndrome is distinguished by a recalcitrant inflammatory state and hematologic disturbances. The acronym VEXAS outlines key disease features: vacuolar anomalies in bone marrow, implication of the E1 enzyme (ubiquitin-like modifier-activating enzyme 1) encoded by UBA1, X-linked inheritance pattern (located on the X chromosome), and an autoinflammatory and somatic presentation (acquired postnatally, non-hereditary).

Epidemiologically, VEXAS syndrome predominantly affects males, with rare cases in females, possibly linked to protective non-mutated genes or acquired X chromosome abnormalities. The diagnosed age range (47–83 years) suggests a later onset or challenges in early detection.

Mechanistically, VEXAS syndrome arises from a somatic mutation in the UBA1 gene, primarily located at the p.Met-41 site. This mutation induces the loss of the cytoplasmic isoform UBA1b and the formation of a catalytically impaired UBA1c subtype, leading to the loss of ubiquitination activity and activation of the innate immune pathway. The mutation predominantly occurs in peripheral blood myeloid cells but not lymphocytes or fibroblasts. The prevalent gene mutation types currently identified encompass the following: p.Met41Thr (c.122T>C), p.Met41Val (c.121A>G), and p.Met41Leu (c.121A>C) (Al-Hakim and Savic 2023). Additional mutations, including splicing site variations, c.167C>T, p.Ser-56Phe have been identified in a subset of VEXAS patients (Poulter et al. 2021). In recent years, as research has progressed, we have uncovered additional genetic variations, such as c.1G>C p.Gly-477Ala and c.1861A>T p.Ser621Cys (Stiburkova et al. 2023; Beck et al. 2023). Continued exploration in this field holds promise for providing critical insights into the underlying disease mechanisms of VEXAS.

**Fig. 1:**
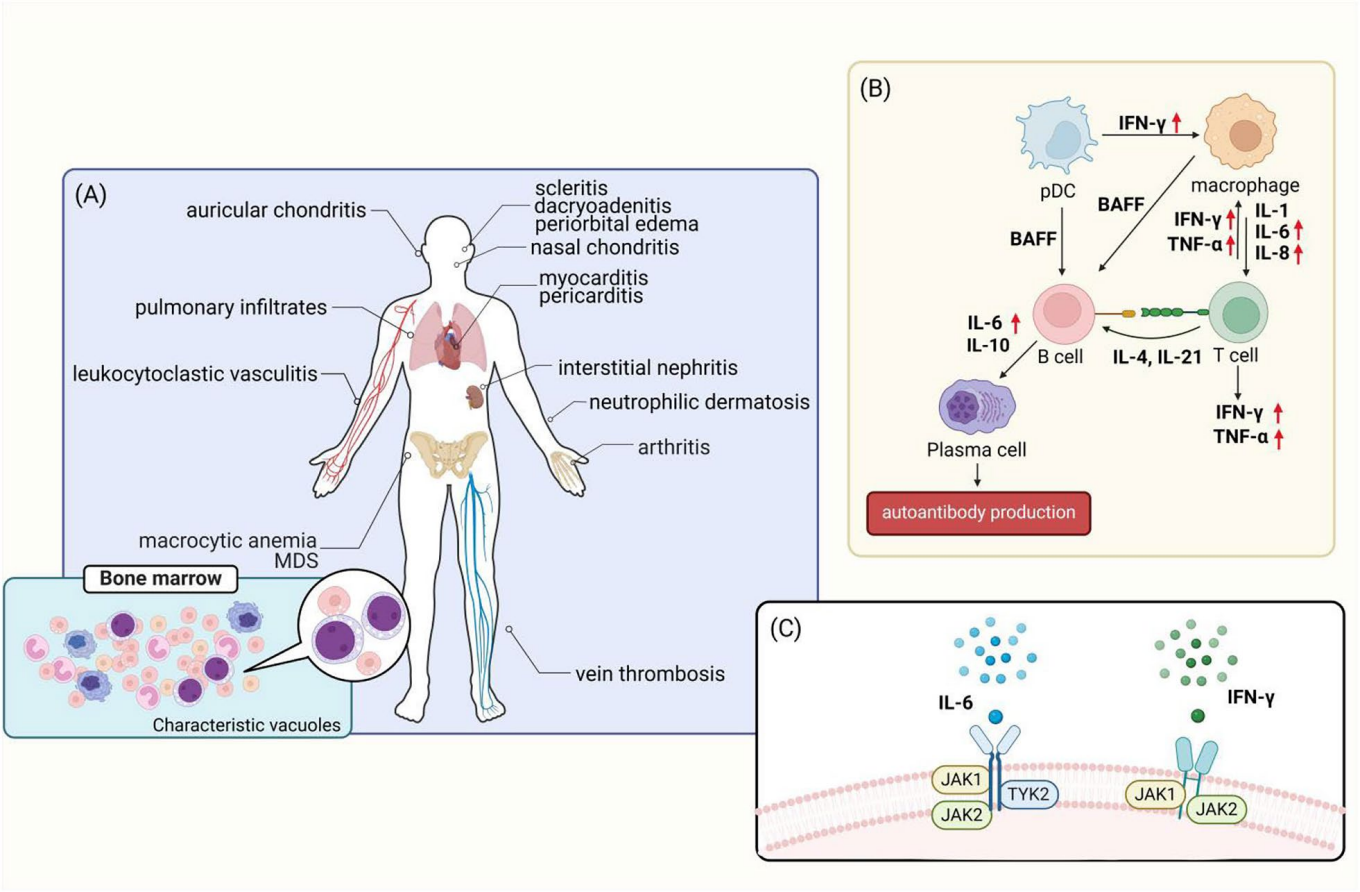
The figure was created with BioRender.com. **A** Common clinical features of VEXAS syndrome include characteristic vacuoles within bone marrow myeloid and erythroid precursor cells. **B** In various autoimmune disorders, plasmacytoid dendritic cells (pDCs) release IFN-γ to stimulate macrophage activation. Subsequently, activated macrophages secrete cytokines such as IL-1, IL-6, and IL-8, fostering T cell activation. Activated T cells, in turn, produce IFN-γ, TNF-α, and other cytokines, enhancing macrophage activation. Both pDCs and macrophages can induce B cell activation by secreting B cell activating factor (BAFF). T cells also contribute to B cell activation through the production of IL-4 and IL-21. Upon activation, B cells produce IL-6 and IL-10, facilitating plasma cell differentiation and autoantibody generation. The red arrows in the figure indicate elevated factors associated with VEXAS syndrome. **C** Factors associated with VEXAS syndrome include IL-6 and IFN-γ, which exert their effects via the JAK-STAT pathway

Symptomatically, within the realm of VEXAS syndrome, the intricate interplay between genetic mutations and dysregulated inflammatory responses contributes to the complex and severe clinical manifestations. The inflammatory manifestations contribute significantly to its clinical presentation. The syndrome induces a broad range of inflammatory responses affecting various organs and systems, leading to symptoms such as fever, pulmonary complications (lung infiltrates, pleural effusion, medium-sized bronchial arterial vasculitis, neutrophilic alveolitis), and dermatological manifestations (neutrophilic dermatosis, tender nodules, sweet syndrome, auricular and nasal chondritis, relapsing polychondritis, nodular vasculitis, giant cell arteritis). Additionally, hematologic abnormalities further characterize the symptomatology of VEXAS syndrome. The syndrome is associated with large-cell anemia, thrombocytopenia, thrombotic diseases, myelodysplastic syndrome (MDS), macrophage activation syndrome (MAS), hemophagocytic lymphohistiocytosis (HLH), and progressive bone marrow failure. These hematologic manifestations contribute to the complexity and severity of the syndrome, posing challenges for early identification and diagnosis (Fig. 1 Created with BioRender.com).

At the forefront of VEXAS syndrome therapeutics, treatment options face limitations without prospective clinical trials. In a retrospective study, “time to next treatment” was employed as an objective marker to assess the effectiveness of different therapeutic strategies. Cytokine-directed therapies, including interleukin-6 (IL-6) inhibitors and Janus kinases (JAK)–signal transducers and activators of the transcription (STAT) inhibitors, demonstrated compelling outcomes. The median time to the next treatment with IL-6 inhibitors was 8 months, whereas no observation of “time to next treatment” was observed during the study with JAK inhibitors (Bourbon et al. 2021). Allogeneic stem cell transplantation emerges as a promising avenue, while recent breakthroughs with ruxolitinib and tocilizumab spark discussions on their potential superiority. This critical mini-review explores JAK inhibitors and IL-6 inhibitors, contributing to the ongoing academic discourse on VEXAS syndrome.

## Genetics and clinical features of VEXAS syndrome

Current extensive cohort studies have provided evidence linking clinical features to specific genetic variants. In a comprehensive study conducted in France, 116 VEXAS patients were categorized into three clusters based on clinical presentations. Patients in cluster 1 exhibit a lower level of inflammation and a mild-to-moderate phenotype. Additionally, this cluster demonstrated a reduced frequency of lung, lymph node, and venous thromboembolism (Georgin-Lavialle et al. 2022). Subsequent genetic testing on patients in cluster 1 revealed a notable prevalence of UBA1 p.Met41Leu mutations within this specific cluster. The UBA1 p.Met41Leu variant was associated with improved survival and a better prognosis.

In another study involving 83 VEXAS patients, a more nuanced understanding of the correlation between genotype and clinical presentation has been attained. Patients harboring the Val variant show a reduced incidence of ear chondritis but an elevated occurrence of periorbital edema and undifferentiated inflammatory syndrome. Furthermore, those carrying the Leu variant exhibit a higher propensity for neutrophilic dermatosis and sweet syndrome. Patients with the Thr variant are more predisposed to inflammatory eye diseases (Ferrada et al. 2022). The study identified 3 factors that independently predict survival in this severe disease: the presence of ear chondritis, development of transfusion-dependent anemia, and p.Met41Val. Ear chondritis is associated with increased survival, while transfusion dependency and the p.Met41Val variant are independently associated with decreased survival. Although MDS is observed in about half of the patients, its presence does not significantly impact mortality (Khitri et al. 2024). The median survival of patients with the Val variant was significantly shorter compared with patients with the Leu or Thr variants. Notably, the Val variant supports less UBA1b translation than either p.Met41Leu or p.Met41Thr, providing a molecular rationale for decreased survival (Ferrada et al. 2022).

## Therapies targeting IL-6 in VEXAS headings

### Mechanism

The gene expression profiles of monocytes and neutrophils in patients were investigated, revealing highly activated inflammatory signaling pathways, including IL-6, tumor necrosis factor (TNF), and interferon-γ (IFN-γ) (Beck et al. 2020). These findings serve as a compelling indicator of the pivotal and integral role of cytokines within the pathophysiological framework of VEXAS. Although there is currently no further research elaborating on the specific mechanistic role of IL-6 in VEXAS syndrome, IL-6 inhibitors can still be considered as a potential therapeutic option.

### Application of tocilizumab in VEXAS

Tocilizumab, a monoclonal antibody that blocks the IL-6 receptor, represents a promising intervention avenue for VEXAS due to its ability to disrupt IL-6-mediated inflammatory processes. Several case reports have cited tocilizumab as a therapeutic option for treating VEXAS. The majority of studies indicate that tocilizumab alleviates the clinical manifestations and inflammatory markers of VEXAS syndrome, reduces steroid dosages and controls anemia (Goyal et al. 2022; Fukuda et al. 2024; Yamaguchi et al. 2023). Additionally, the efficacy of tocilizumab is not limited to a single genotype. Cases of tocilizumab use have been reported in patients with nearly all genotypes, demonstrating varying degrees of therapeutic effectiveness (Table 1). Nevertheless, in certain instances, tocilizumab proves ineffective in controlling anemia and joint pain (Made et al. 2022).

**Table 1:** Summary of treatment outcomes and adverse events in VEXAS syndrome mutations

Mutation	Complication	Treatment	Outcome	Adverse event	Refs.
p.Met41 Thr (c.122T>C)		IL-6Ri			
	Anemia Cellulitis Idiopathic orbital myositis Lung involvement Sweet syndrome	1 tocilizumab 162 mg/week	Transfusion independent Skin lesions cleared Corticosteroids decreased	No	Goyal et al. (2022)
	Blepharitis Chondritis Leukocytoclastic vasculitis Macrocytic anemia Oligoarthritis Renal insufficiency RP Skin lesions	2 tocilizumab 162 mg/week	One patient’s renal dysfunction is now managed, but anemia and joint pain persist One patient has anemia and inflammation improved	No	Made et al. (2022)
	Macrocytic anemia MDS Vasculitis RP	1 tocilizumab 8 mg/kg/month	Resulted in a quiescent disease state for 1.5 years	Discontinuation of tocilizumab due to recurrent infections	Made et al. (2022)
	Macrocytic anemia RP Skin rash	1 tocilizumab 162 mg/week to 162 mg/10 days	PSL dosage and inflammatory parameters (CRP, WBC) decreased Inflammatory symptoms and anemia improved	Leukopenia	Kunishita et al. (2022)
	Meningitis Peritonitis Pericarditis RP Scleritis Skin rash	1 tocilizumab 8 mg/kg/4 weeks	PSL dosage and inflammatory parameters (CRP, WBC) decreased Inflammatory symptoms improved	Herpes zoster Cellulitis Skin ulceration	Kunishita et al. (2022)
	Cellulitis GCA Nodular erythema Polymyalgia rheumatica Renal insufficiency RP	1 tocilizumab 8 mg/kg/month	PSL dosages decreased Inflammation was controlled Skin lesions decreased	No	Fukuda et al. (2024)
	Auricular chondritis Episcleritis Lung involvement MDS Nodular erythema Periorbital edema	1 tocilizumab 480 mg/month	Inflammatory symptoms improved, and no inflammatory crises were observed	No	Fanlo et al. (2023)
	BHL Cutaneous polyarteritis nodosa Neutrophilic dermatosis Nodular erythema	1 tocilizumab 400 mg/month	PSL dosages decreased Inflammation was controlled The level of oxidative stress marker d-ROMs decreased	No	Tozaki et al. (2022)
	Chondritis Macrocytic anemia MDS PCM Vasculitis	5 tocilizumab	All patients have CRP levels and hormone dosage reduced but are unable to achieve long-term symptom control, still have progressive cytopenia	Four patients discontinued the medication due to uncontrolled symptoms	Koster et al. (2021)
p.Met41 Thr (c.122T>C)		JAKi			
	cPAN HLH-MAS Lung involvement Lymphocytopenia	1 ruxolitinib 15 mg TD	Clinical symptoms were significantly improved	Died of sepsis	Kao et al. (2022)
	DVT Lung involvement Macrocytic anemia Skin rash Vasculitis	1 upadacitinib 15 mg/d	Prednisone dosage and level of CRP decreased Clinical symptoms resolved	No	Muratore et al. (2022)
	Lung involvement Macrocytic anemia MDS-MLD Neutrophilic dermatosis Polyarthritis	1 tofacitinib 20 mg/day	No clinical benefit	No	Lötscher et al. (2021)
	Anemia Auricular chondritis DVT Lung​ involvement Neutrophilic​ dermatosis Sweet syndrome	1 tofacitinib 5 mg TD	Symptoms resolved Inflammatory markers and cytopenias normalized	Secondary drug failure occurred after 3 months	Salehi et al. (2023)
	MDS	1 baricitinib	No significant remission, and still have RBC transfusion dependence	No	Islam et al. (2022)
p.Met41 Val (c.121A>G)		IL-6Ri			
	Anterior uveitis DVT Eosinophilic vasculitis with perivascular dermatitis Lung involvement Macrocytic anemia Raynaud syndrome Skin rash	2 tocilizumab 162 mg/week	One patient has hormone dosage reduced, but symptoms relapsed, died One patient has anemia improved, but there was no change in clinical symptoms or inflammatory markers	One patient died of jejunum perforation	Made et al. (2022)
	Axonal polyneuropathy Lung involvement Macrocytic anemia Skin rash	1 tocilizumab 162 mg/week to 8mg/kg/month	Died	Died of ileum perforation	Made et al. (2022)
p.Met41 Val (c.121A>G)		JAKi			
	Dacryoadenitis MDS Pulmonary fibrosis	1 tofacitinib 5 mg TD	Orbital and systemic inflammatory symptoms improved	No	Beecher et al. (2022)
	Leukocytoclastic vasculitis Lung involvement	1 tofacitinib 5 mg TD	Not described	Not described	Habershon et al. (2022)
	Anemia Dacryoadenitis DVT Lung​involvement Neutrophilic dermatosis	2 tofacitinib 5 mg TD	PSL dosage decreased Ameliorated​ disease-related​ symptoms Resolved​ cytopenias Improved​ inflammatory ​markers	No	Salehi et al. (2023)
p.Met41 Leu (c.121A>C)		IL-6Ri			
	Anemia Arteriovenous fistula Hypergamma globulinaemia Iliac artery aneurysm Neutrophilic dermatosis	1 tocilizumab 162 mg/week	Recurrent fever and elevated CRP levels improved	No	Yamaguchi et al. (2023)
	DVT GCA Lung involvement RP Scleritis Skin rash	1 tocilizumab 162 mg/week to 8 mg/kg/2 weeks	PSL dosage and inflammatory parameters (CRP, WBC) decreased Inflammatory symptoms and anemia improved Arthritis, skin rash, and transfusion dependence were not fully controlled	Herpes zoster	Kunishita et al. (2022)
p.Met41 Leu (c.121A>C)		JAKi			
	Skin rash	1 tofacitinib 5 mg TD	Cutaneous lesions significantly improved Tapered off prednisone	No	Fahmy et al. (2023)
c.118-1 G>C		IL-6Ri			
	Arthritis Lung involvement Macrocytic anemia MACROPHAGE activation syndrome skin rash	1 tocilizumab	No significant improvement	Infusion reaction	Miyoshi et al. 2023)
No detailed described					
p.Met41 Thr(c.122T>C) p.Met41 Val(c.121A>G) p.Met41 Leu(c.121A>C) Splice mutations	Chondritis Lung involvement Macrocytic anemia MDS Venous thromboembolism	6 tocilizumab	All patients respond well with symptom control (cough or dyspnea) and resolution of imaging abnormalities in the lung	Not described	Moura et al. (2023)
p.Met41 Thr(c.122T>C) p.Met41 Val(c.121A>G) p.Met41 Leu(c.121A>C) c.118-1G>C	Lung involvement MDS MGUS Skin lesions Thrombophlebitis	15 tocilizumab 8 mg/kg/3–4 weeks	Nine patients had prednisone dosages decreased and hemoglobin levels increased Nine patients had inflammatory symptoms improved, and CRP levels decreased Three patients showed a decrease in UBA1 level	All patients still experience progressive marrow failure One patient died in a hyperinflammatory episode One patient died of cerebral infarction	Johansen et al. (2023)
p.Met41 Thr(c.122T > C) p.Met41 Val(c.121A > G) p.Met41 Leu(c.121A > C) Splice mutations	Chondritis Lung involvement Macrocytic anemia MDS Venous thromboembolism	1 ruxolitinib 2 tofacitinib 2 baricitinib 2 upadacitinib	Respond well with symptom control (cough or dyspnea) and resolution of imaging abnormalities	Not described	Moura et al. (2023)
p.Met41 Thr(c.122T>C) p.Met41 Val(c.121A>G) p.Met41 Leu(c.121A>C) c.118-1G>C c.118-2T>C	MDS Monoclonal gammopathy	12 ruxolitinib 5 mg-20 mg TD 11 tofacitinib 4 baricitinib 3 upadacitinib	Overall: 3-month clinical response rate: 57.1% 3-month biological response rate: 53.6%Ruxolitinib: 1-month clinical response rate: 67% 6-month clinical response rate: 87% Other JAKi: 1-month clinical response rate 38% 6-month clinical response rate 11%	Overall: Infection (36.7) Thromboembolic complications (20%) Tofacitinib: one patient died of legionellosis Ruxolitinib: Two patients receiving experienced progression MDS. One patient died of colon cancer progression, one patient died of an undetermined cause	Heiblig et al. 2022)

*BHL* bilateral hilar lymphadenopathy, *cPAN* cutaneous polyarteritis nodosa, *CRP* C-reactive protein, *DVT* deep vein thrombosis, *d-ROMs* derivatives of reactive oxygen metabolites, *GCA* giant-cell arteritis, *HLH-MAS* hemophagocytic lymphohistiocytosis-associated macrophage activation syndrome, *IL-6Ri* interleukin-6 receptor inhibitor, *JAKi* janus kinase inhibitor, *MDS* myelodysplastic syndrome, *MGUS* monoclonal gammopathy of unknown significance, *MLD* multilineage dysplasia, *PCM* plasma-cell myeloma, *PSL* prednisolone, *RP* relapsing polychondritis, *TD* twice daily

### Selection of dosage forms

There are no clear guidelines or standards for the dosage form of tocilizumab in the treatment of VEXAS syndrome, and current treatments are largely empirical. The dosages detailed in Table 1, including 162 mg/week subcutaneously and 8 mg/kg/month intravenously, are derived from protocols for other conditions. Comparative data indicate that patients receiving subcutaneous injections generally experience fewer adverse reactions. However, it is notable that the two recorded fatalities occurred in the subcutaneous treatment group, underscoring the need for a thorough evaluation of the benefits and risks associated with these two primary administration methods and dosages. Importantly, both deceased patients were p.Met41 Val-positive, an independent risk factor for decreased survival in VEXAS patients. This raises the question of whether the administration method and dosage of tocilizumab impact patient outcomes and survival rates, especially for p.Met41 Val-positive patients, necessitating careful consideration in treatment choices. Given this information, the 162 mg/week subcutaneous dosage of tocilizumab might be preferentially recommended for VEXAS patients. However, the current data on the use of tocilizumab in VEXAS syndrome are limited. Comprehensive, large-scale studies are needed to determine the efficacy, optimal administration methods, and appropriate dosages of tocilizumab and to refine treatment strategies for VEXAS patients with different genetic profiles.

### Limitations and future prospects

During tocilizumab therapy, adverse reactions may occur. Some patients discontinue treatment due to uncontrolled symptoms or infusion reactions during the therapeutic process (Koster et al. 2021; Miyoshi et al. 2023). The most severe adverse events observed were two cases of intestinal perforation in patients receiving tocilizumab treatment (Made et al. 2022), however, no such severe adverse reaction was observed in subsequent studies. Notably, both of these patients were p.Met41 Val-positive, a known independent risk factor for mortality in VEXAS patients. Therefore, it remains unclear whether tocilizumab treatment directly contributed to their deaths.

At the same time, during treatment with tocilizumab, there have been cases of symptom relapse following steroid tapering, however, this necessitates a comprehensive assessment of individual patient profiles and underlying therapeutic considerations. In this case report, the patient experiencing relapse exhibited a prolonged interval from symptom onset to diagnosis in comparison to other cases (Kunishita et al. 2022), delay in initiating timely therapeutic intervention, emphasizing the critical importance of early identification and management of VEXAS syndrome, which holds promise for favorable therapeutic responses and prognostic outcomes.

There are no established guidelines for the selection of therapeutic agents in the treatment of VEXAS syndrome. Many studies summarized in this paper lack detailed explanations for the use of tocilizumab. Only one case report extensively discussed the selection of tocilizumab based on elevated serum IL-6 levels in patients, which resulted in a positive response, significantly reducing inflammatory symptoms and steroid dosage (Goyal et al. 2022). This highlights the necessity of tailoring treatment approaches according to the inflammatory cytokine levels in patients’ serum, particularly advocating the use of tocilizumab in cases with significantly elevated IL-6 levels.

Given that VEXAS syndrome is caused by genetic mutations, selecting different therapeutic approaches based on genotype is also crucial and clinically valuable. Table 1 in this review summarizes the responses of patients with different genotypes to various treatments and dosages. Current data suggest that p.Met41 Thr-positive patients may respond better to tocilizumab compared to JAK inhibitors. Although these results may be limited by sample size, they indicate a potential direction for exploring treatment options for VEXAS patients with different genotypes.

## Therapies Targeting JAK/STAT in VEXAS

### Mechanism

The Janus kinases (JAK)–signal transducers and activators of the transcription (STAT) pathway have a pivotal role in autoimmunity and systemic inflammation (Xin et al. 2020). Numerous inflammation-related cytokines exert their effects through this pathway, including ILs, TNFs, granulocyte–macrophage colony-stimulating factors, and IFN-γ (Morris et al. 2018), the present serological evidence concerning VEXAS syndrome indicates elevated levels of cell factors, notably IL-6 and IFN-γ, which are modulated via the JAK pathway. This observation offers a theoretical underpinning for the potential application of JAK inhibitors.

### Application of JAK inhibitors

Currently, several JAK inhibitors, such as ruxolitinib, tofacitinib, baricitinib, and upadacitinib, have been utilized in the treatment of VEXAS (Bindoli et al. 2023). However, due to the recent diagnosis of VEXAS in 2020 and the intricate clinical presentations leading to patients being dispersed across various medical disciplines, there is a lack of systematic diagnosis, treatment, and data documentation for many VEXAS cases. Therefore, evaluations of the therapeutic efficacy of JAK inhibitors in VEXAS often rely on retrospective studies for comprehensive insights. In a retrospective series involving 11 VEXAS patients, treatment outcomes were evaluated based on the time to the addition of a new steroid-sparing agent (“time to next treatment”). The JAK treatment group did not reach the “time to next treatment “during the study period (Bourbon et al. 2021). In several other studies and case reports, patients treated with JAK inhibitors exhibited improved clinical symptoms and favorable changes in laboratory indicators (Moura et al. 2023; Muratore et al. 2022; Salehi et al. 2023). In a recent retrospective study involving 30 patients receiving JAK inhibitor therapy, it was noted that ruxolitinib demonstrated superior clinical efficacy rates at 1 and 6 months, reaching 67% and 87%, respectively. In contrast, other JAK inhibitors exhibited lower clinical efficacy rates, with percentages of 38% and 11% at the corresponding time points (Heiblig et al. 2022). In certain cases, alternative JAK inhibitors, such as tofacitinib, have demonstrated efficacy in ameliorating inflammatory symptoms and reducing corticosteroid dosage (Salehi et al. 2023; Beecher et al. 2022; Fahmy et al. 2023). This observation suggests that JAK inhibitors, especially ruxolitinib, serve as an effective therapeutic approach for VEXAS.

### Selection of dosage forms

Similarly, the optimal dosage of JAK inhibitors for patients with VEXAS syndrome has yet to be established. According to the study by Heiblig et al., ruxolitinib has a wide therapeutic window, with doses ranging from 15 to 25.4 mg/day demonstrating efficacy without severe adverse effects. It is recommended to initiate treatment at a dose of 5–10 mg twice daily (TD) in elderly patients or those with neutropenia, adjusting based on clinical and biological response dynamics, with a maximum dose of 20 mg TD (Heiblig et al. 2022). However, due to hematological safety concerns associated with ruxolitinib, including thrombocytopenia, anemia, and neutropenia, it may not be suitable for patients with severe cytopenia, particularly those with severe neutropenia.

The commonly used dose of tofacitinib for other immune diseases is 5 mg TD. As summarized in Table 1 of this review, most studies also chose a dosage of 5 mg TD, indicating a favorable therapeutic effect. However, one study used a dose of 20 mg/day without observing significant efficacy. This raises the question of whether 5 mg TD might be the effective or optimal dose for treating VEXAS with tofacitinib. Notably, the patient treated with 20 mg/day had more severe multisystem involvement but did not show significant improvement with the higher dose. This suggests that increasing the dose with disease severity may not be an effective strategy. However, as this review includes only one such case, the conclusion is not definitive and highlights the need for more research and larger sample sizes to further explore tofacitinib’s efficacy. Due to their infrequent use, other JAK inhibitors have variable efficacy and unpredictable adverse effects. As a result, the optimal therapeutic dosage and suitable patient profiles for these inhibitors remain unclear at present.

### Limitations and future prospects

While a majority of patients receiving JAK inhibitor therapy demonstrate a reduction in clinical symptoms and inflammatory markers, improvements in anemia are observed in only a minority of cases. Notably, within the study, two cases of MDS progression were still observed in two individuals treated with ruxolitinib (Heiblig et al. 2022). Whether JAK inhibitors can effectively manage the progressive hematologic manifestations in VEXAS patients remains a topic of ongoing debate. Based on previous research findings, there is a suggestion that the administration of the JAK inhibitor increases the risk of thrombosis in patients, with this risk escalating with dosage increments (Ytterberg et al. 2022). Moreover, tofacitinib and baricitinib have been implicated in potentially elevating the risk of venous thromboembolism (VTE) (Scott et al. 2018). However, a comprehensive meta-analysis of 42 studies did not find supporting evidence for a higher VTE risk in patients treated with JAK inhibitors (Yates et al. 2021). Considering that the incidence of venous thrombosis among VEXAS patients is approximately 40% (Groarke et al. 2021), patients undergoing JAK inhibitor therapy should remain vigilant in managing thrombotic events. Recently, in VEXAS patients with the p.Met41Val mutation and undergoing JAK inhibitor treatment, a slightly elevated occurrence of severe infections was observed (Valence et al. 2023). This indicates the need for careful consideration when choosing therapeutic drugs for patients with the p.Met41Val variant, emphasizing caution in the use of JAK inhibitors and careful attention to the concurrent administration of anti-infective medications.

The current literature on VEXAS syndrome is sparse, consisting primarily of case reports and clinical studies that often lack comprehensive data on patient genotypes and specific drug dosages. This paucity of detailed information hinders the determination of optimal dosages for various JAK inhibitors and complicates the selection of appropriate inhibitors for patients with differing genotypes. Therefore, extensive large-scale clinical studies are essential to elucidate these aspects.

## Discussion

The treatment landscape for VEXAS syndrome remains largely uncharted, lacking established guidelines or optimal therapeutic protocols. While several drugs are currently under investigation, such as Azacytidine, a DNA methyltransferase inhibitor (DNMTI), this review primarily focuses on IL-6 inhibitors and JAK inhibitors.

JAK inhibitors have demonstrated promise in various studies, exhibiting a broad therapeutic window. For example, ruxolitinib has been effectively utilized in clinical practice, allowing for convenient dosage adjustments during treatment. However, it is important to note that adverse reactions, particularly infections and VTE, are frequently observed in clinical settings. This underscores the necessity of implementing preventive measures when utilizing JAK inhibitors.

IL-6 inhibitors have shown promising therapeutic effects in current clinical applications. However, the optimal timing for initiating IL-6 inhibitor treatment and identifying the appropriate patient population remain areas for further investigation. Currently, it is recommended to use IL-6 inhibitors in patients with elevated serum IL-6 levels to optimize treatment precision. For p.Met41Thr-positive patients, IL-6 inhibitors such as tocilizumab are also recommended, starting with a subcutaneous dose of 162 mg per week. Due to the occurrence of two fatal cases of bowel perforation in patients treated with tocilizumab, and considering that VEXAS patients require long-term use of glucocorticoids, which inherently increases the risk of gastrointestinal complications, it is crucial to monitor and evaluate the gastrointestinal status of patients during tocilizumab treatment.

Current research on VEXAS syndrome reveals numerous unresolved questions regarding the clinical use of IL-6 inhibitors and JAK inhibitors. It has been confirmed that clinical manifestations vary among patients with different genotypes. Therefore, studying the impact of different treatments on various genotypes is a crucial step for advancing VEXAS therapy. Table 1 in this paper categorizes existing studies on the use of IL-6 and JAK inhibitors in VEXAS treatment by genotype to better understand the effectiveness of different treatments. Notably, patients with the Val variant have a lower median survival rate compared to other genotypes, and their risk of severe infections is significantly increased. Additionally, there have been two fatal cases of bowel perforation, both occurring in Val-positive patients. Therefore, when selecting treatment options for Val-positive patients, it is essential to exercise caution and thoroughly consider the associated risks and benefits, with the current recommendation against prioritizing subcutaneous tocilizumab. For Thr-positive patients, initial treatment with tocilizumab at a dose of 162 mg/week appears promising, but this approach requires validation through larger cohort studies. While IL-6 inhibitors and JAK inhibitors appear effective in controlling inflammation, as evidenced by the improvement in symptoms such as skin lesions and edema, as well as laboratory indicators, they seem less effective in addressing the hematological involvement in VEXAS syndrome. Many patients remain dependent on transfusions and require combination therapy with other medications.

VEXAS represents a newly identified hematoinflammatory syndrome arising from a somatic mutation in the *UBA1* gene. Clinicians must maintain vigilance when faced with patients exhibiting concurrent hematologic and inflammatory symptoms, striving for early diagnosis and genotype identification to enable timely intervention. Investigating whether distinct genotypes result in varied clinical presentations among VEXAS syndrome patients, along with the necessity for adjusting clinical medication appropriately based on genotypes and clinical manifestations, demands thorough research within the clinical domain.

## Data Availability

The data and materials presented in this manuscript are derived exclusively from published sources. The figure was created with BioRender.com.

## Abbreviations

DNMTI: DNA methyltransferase inhibitor
HLH: Hemophagocytic lymphohistiocytosis
MAS: Macrophage activation syndrome
MDS: Myelodysplastic syndrome
IFN-γ: Interferon-γ
IL-6: Interleukin-6
JAK: Janus kinases
STAT: Signal transducer and activator of transcription
TD: Twice daily
TNF: Tumor necrosis factor
VTE: Venous thromboembolism
